# Sex differences in brain atrophy in dementia with Lewy bodies

**DOI:** 10.21203/rs.3.rs-2516427/v1

**Published:** 2023-01-27

**Authors:** Javier Oltra, Annegret Habich, Christopher G. Schwarz, Zuzana Nedelska, Scott A. Przybelski, Anna Inguanzo, Patricia Diaz-Galvan, Val J. Lowe, Ketil Oppedal, Frederic Blanc, Afina W. Lemstra, Jakub Hort, Alessandro Padovani, Irena Rektorova, Laura Bonanni, Federico Massa, Milica G. Kramberge, John-Paul Taylor, Jon Snædal, Zuzana Walker, Angelo Antonini, Barbara Segura, Carme Junque, Eric Westman, Bradley F. Boeve, Dag Aarsland, Kejal Kantarci, Daniel Ferreira

**Affiliations:** University of Barcelona; Karolinska Insitutet; Mayo Clinic and Foundation; Charles University; Mayo Clinic and Foundation; Karolinska Insitutet; Mayo Clinic and Foundation; Mayo Clinic and Foundation; University of Stavanger; University Hospital of Strasbourg; VU University; Charles University; University of Brescia; Masaryk University; University of Chieti – Pescara; University of Genoa; Ljubljana University; Newcastle University; Landspitali University Hospital; University College London; University of Padua; University of Barcelona; University of Barcelona; Karolinska Institutet; Mayo Clinic and Foundation; King’s College London; Mayo Clinic and Foundation; Karolisnka Institutet

**Keywords:** Atrophy, MRI, dementia with Lewy bodies, sex differences

## Abstract

**Background and objectives.:**

Sex is an important contributing factor to neuroimaging phenotypes in brain disorders. However, little is known about the contribution of sex differences to the neurodegeneration in dementia with Lewy bodies (DLB). We investigated sex differences in probable DLB patients by using both visual rating scales of lobar atrophy and automated estimations of regional atrophy.

**Methods.:**

We included 442 probable DLB patients from the European-DLB consortium and the Mayo Clinic who have magnetic resonance imaging (MRI) data available. We assessed sex differences and the sex-by-age interaction in two largely independent samples through visual rating scales of lobar atrophy (n = 333; mean age 73 ± 8 years, 62% males) and automated regional estimations of gray matter (GM) volume and mean cortical thickness (CTh) (n = 165; mean age 69 ± 9 years, 72% males). We used binary logistic regression and ANOVA for statistical analysis.

**Results.:**

We found a statistically significantly higher likelihood of frontal atrophy measured by the global cortical atrophy-frontal subscale (GCA-F) in males (40% of males had an abnormal GCA-F score versus 29% of females, *P*-value = 0.006). Using automated estimations, we found smaller GM volumes in 6 cortical regions in males compared with females, as well as smaller GM volume in the entorhinal cortex and thinner olfactory cortices in females, compared with males. The sex-by-age interaction showed statistically significant results in 6 cortical volumes and 7 mean CTh estimations (*P*-value ≤ 0.05), accentuated in the right middle frontal gyrus (FDR-adjusted *P*-value = 0.047). These cross-sectional interactions indicated that while females have statistically significantly less atrophy than males at younger ages, differences become non-significant at older ages, with females showing the same level of atrophy than males around the age of 75.

**Conclusions.:**

This study demonstrates sex differences on brain atrophy in probable DLB. While male DLB patients have a more widespread pattern of cortical atrophy at younger ages, these sex differences tend to disappear with increasing age. Longitudinal studies will help establish these cross-sectional findings and inform on sex and age considerations to the use of MRI in clinical routine, as the field moves towards precision medicine.

## Introduction

Recently, the influence of sex and gender on neurodegenerative diseases, especially Alzheimer’s disease (1) and Parkinson’s disease (2) has been spotlighted. Sex and gender differences are central to current precision medicine approaches. They are expected to play a relevant role in the prevention, diagnosis, and treatment of neurodegenerative diseases in the upcoming years (3, 4). However, very few studies have investigated sex differences in dementia with Lewy bodies (DLB), the second most common form of neurodegenerative dementia. Particularly, neuroimaging studies on sex differences in DLB are scarce, despite sex is a defining feature of neuroimaging phenotypes in brain disorders (5).

Although DLB is considered a predominantly male disease, the sex ratio in DLB varies depending on the cohort. For example, the female-to-male ratio ranges from 0.59:1 in the Swedish Dementia Registry (SveDem) (6) to 0.81:1 in data from secondary care services from the UK (7) and 1.20:1 in the French National Alzheimer database (BNA) (8).

Regarding clinical diagnosis, several studies have reported sex differences in three out of the four core clinical DLB features. In particular, some studies reported a higher likelihood of parkinsonism and rapid eye movement sleep behavior disorder (RBD) in male DLB patients (9, 10). Concerning visual hallucinations, some studies reported a higher frequency in female DLB patients (11, 12), while other studies reported so for male DLB patients (10). No sex differences were reported for cognitive fluctuations.

A recent neuropathological study found that male DLB donors more frequently have “pure” Lewy body pathology (13). In contrast, a previous report did not show differences between male and females in the spread of Lewy body pathology (14). Furthermore, female DLB donors are more likely to have brain co-pathologies such as Alzheimer’s and cerebrovascular diseases (13). The higher prevalence of Alzheimer’s disease co-pathology observed in DLB females in postmortem studies has also been supported in vivo with the use of β-amyloid and tau cerebrospinal fluid biomarkers (12, 15).

Unfortunately, the data on potential sex differences in magnetic resonance imaging (MRI) measures of neurodegeneration in DLB is limited to only two previous studies, to the best of our knowledge. A study from 2004 investigated sex differences in regional cortical gray matter (GM) volume in 8 male and 8 female DLB patients (16). Male DLB patients had lower GM volume than females in the right dorsal frontal and bilateral parietal cortices. In a recent multicenter study on 86 DLB patients (49 males and 37 females) from the European DLB (E-DLB) consortium (17), we found that frontal atrophy as assessed on visual ratings was associated with the male sex and older age in DLB (18).

MRI studies in DLB are usually small, which makes it difficult to reach sufficient statistical power, especially for the female patient group which is often even smaller in sample size. This likely explains the lack of MRI studies on sex differences in DLB, despite the current interest in the topic. We overcame this limitation by leveraging a large multicenter MRI cohort of 442 probable DLB patients, the largest cohort of this type in the field. The cohort included 280 male and 162 female DLB patients. Our main aim was to investigate sex differences in measures of neurodegeneration using MRI, through two different methods: visual ratings of brain atrophy and automated methods for volumetric and cortical thickness measures. The main reason for using two methods was to generate knowledge directly applicable to clinical settings (visual ratings from radiologists), at the same time that we replicated and expanded radiological findings using more sensitive automated techniques for regional brain atrophy. Further, this approach served to validate the findings and generalize across common MRI techniques. To achieve this, we divided the cohort in two partially independent samples. For visual ratings we assessed a sample of 333 DLB patients (205 males and 128 females) with clinical MRI scans of varied quality. For volumetric and cortical thickness measures we assessed 165 DLB patients (119 males and 46 females; 56 of them shared with the 333 sample) with research-oriented high-resolution MRI scans. We hypothesized that male DLB patients would have more frontoparietal atrophy, based on the two previous studies (16, 18). Further, we anticipated that female DLB patients would have more GM atrophy in the medial temporal lobe, based on the more prevalent Alzheimer’s disease co-pathology in female DLB patients (13). We also had a strong interest in investigating the interaction between sex and age with regard MRI measures because our previous study informed on the combined effect of both sex and age on frontal atrophy in DLB (18). Hence, we hypothesized that DLB patients would show a sex-by-age interaction in frontal regions.

## Methods

### Participants

This study includes patients from multiple centers from the European DLB consortium (E-DLB, https://www.e-dlb.com/) (17) and the Mayo Clinic in the U.S. (19). First, we assessed sex differences using visual rating scales on clinical MRIs from 333 DLB patients (205 males and 128 females) from 15 E-DLB centers (more detail in Supplementary Table 1). Second, we assessed sex differences using research-oriented automated methods on high-resolution MRIs from 165 DLB patients (119 males and 46 females) from three E-DLB centers (n = 97) and the Mayo Clinic cohort from Rochester, MN, U.S. (n = 68). (Supplementary Table 1).

DLB diagnosis was done following the 2005 international consensus criteria for probable DLB (20). The presence of core clinical features was collected, including parkinsonism, visual hallucinations, cognitive fluctuations, and clinical history of probable RBD. Exclusion criteria were: (i) presence of acute delirium; (ii) terminal illness; (iii) previous stroke; (iv) psychotic or bipolar disorder; (v) craniocerebral trauma; and (vi) recent diagnosis of significant systemic disease. Age and years of education were collected for statistical analysis, and the Mini-Mental State Examination (MMSE) was used as a measure of global cognitive performance.

Alzheimer’s disease co-pathology was assessed through positivity in β-amyloid and tau biomarkers. EDLB centers used cerebrospinal fluid (CSF) β-amyloid 1–42 and phosphorylated tau biomarkers, while the Mayo Clinic used positron emission tomography (PET) Pittsburgh compound B (PiB) and Flortaucipir (18F-AV-1451). CSF and PET biomarkers were combined as done before (15), and biomarker levels were classified into normal or abnormal based on center-specific established cut-points, as explained in prior publications (15, 19).

For white matter hyperintensity (WMH) burden as a common biomarker of cerebrovascular disease, we used both the Fazekas scale (21) and a semi-automated method for WMH volume estimation, which is fully described elsewhere (22, 23).

The ethics committee of each center approved the data collection. All patients or appropriate surrogates gave written consent to their participation in the study.

### MRI acquisition

The MRI scans were acquired using 1.5 and 3 T scanners, including a high-resolution 3D T1-weighted magnetization prepared rapid gradient echo (MPRAGE) sequence and a fluid-attenuated inversion recovery (FLAIR) sequence, as described in more detail in previous publications (22, 24).

### MRI visual assessment and automated preprocessing

MRI scans of the clinical cohort were rated centrally at Karolinska Institutet by a single experienced neuroradiologist, who had previously demonstrated excellent intra-rater and inter-rater reliability (25). Lobar atrophy was assessed with three visual rating scales based on T1-weighted images (26). Frontal lobe atrophy was assessed with the global cortical atrophy-frontal subscale (GCA-F) (27), medial temporal lobe atrophy with the medial temporal atrophy (MTA) scale (28); and posterior cortex atrophy with the posterior atrophy (PA) scale (29). Visual ratings were classified into normal/abnormal using established cutoffs (26). In the case of the MTA scale, both age-adjusted and unadjusted scores were used, depending on the statistical analysis as explained below. Since age corrections for GCA-F and PA do not improve their diagnostic performance, unadjusted scores were used (26). Procedures and methods are described in detail in previous publications (21, 26).

Regarding the research-oriented method for automated estimation of regional volume and cortical thickness, preprocessing was performed as detailed previously (22). Briefly, image processing was performed centrally at the Mayo Clinic using Advanced Normalization Tools (ANTs) (30). The Mayo Clinic Adult Lifespan Template (MCALT, https://www.nitrc.org/projects/mcalt/) was used to estimate volumes and mean cortical thickness, with MCALT tissue priors and settings (31). Altogether, 82 cortical, 12 subcortical, and 2 brainstem regions of interest (ROIs) were estimated (see ROIs in Supplementary Table 2). The unified segmentation algorithm in SPM12 (Wellcome Trust Center for Neuroimaging, London, UK) was used for volume estimation. Next, ANTs DiReCT was used for the mean cortical thickness estimation of the cortical ROIs from the tissue probabilities (32). Moreover, the estimated total intracranial volume was calculated from the tissue probabilities.

Using multiple linear regression models, volume and cortical thickness of each ROI were corrected for confounding variables and the residuals obtained from the regression models were used for statistical analysis. More specifically, for analyses on the effect of sex, the residuals were obtained from a model with center and age as the predictors (*model 1*). For analyses investigating the interaction between sex and age, the residuals were obtained from a model with center as the only predictor (*model 2*). The estimated total intracranial volume was included as an additional predictor in the models 1 and 2 with GM volumes (but not with cortical thickness) as the dependent variable. Supplementary models 1 and 2 including MMSE as an extra predictor were fitted for sensitivity analyses.

### Statistical analyses

All the analyses were performed using R (The R Foundation for Statistical Computing; version 4.1.0).

Differences in demographic and clinical variables and biomarkers were assessed by t-test for continuous variables, Mann–Whitney U test for ordinal variables, and the Pearson’s chi-squared test or the Fisher’s exact test for categorical variables.

Regarding the clinical cohort, visual rating scales were analyzed using two types of binary logistic regression models for dichotomized variables as the outcome (0, normal visual rating score; 1, abnormal visual rating score). The first model tested for the effect of sex while controlling for the age effect. The second model tested for the interaction between sex and age. For the MTA scale, age-adjusted scores were used to test for the effect of sex; and the unadjusted scores were used to test for the interaction between sex and age.

Regarding the research-oriented automated method, two series of analyses of variance (ANOVA) were performed separately on volume and cortical thickness estimations. In each model, each ROI served as the dependent variable. The first series of models consisted of one-way models with sex as the independent variable. The second series of models consisted of two-way models with sex, age, and the interaction between sex and age as the independent variables. Cohen’s d was used to estimate effect sizes for the one-way models, and ηp^2^ for the two-way models. For ROI analyses on volume and cortical thickness, we report uncorrected *P*-values followed by false discovery rate (FDR) (33) adjusted *P*-values within type of measure (GM volume or cortical thickness) and model (one-way or two-way).

Additionally, we followed main analyses with three further one-way ANOVAs to test whether our sex findings were independent of AD co-pathology, *APOE* genotype, and WMH burden. These analyses were limited to the ROIs showing significant sex differences in the main analyses. For WMH burden and *APOE* genotype, we compared DLB males and DLB females on new residuals calculated using WMH burden or *APOE* genotype as extra predictors, separately. This approach was not feasible for AD co-pathology due to the limited group size of females with a positive AD biomarker. Hence, we replicated the main one-way ANOVA in the subsample of DLB patients with Alzheimer’s disease biomarker available (n = 122), and then the significant findings in the subsample of male and female DLB patients with a negative Alzheimer’s disease biomarker (n = 109).

The significance level was set at *P*-value ≤ 0.05 in all statistical models.

## Results

### Sociodemographic and clinical characteristics

Table 1 shows that there were no statistically significant differences between probable DLB males and females in most of the demographic and clinical variables. Nonetheless, in the clinical cohort, DLB females were older than DLB males. In the research-oriented cohort, DLB males had a lower MMSE score than DLB females, and DLB males had a higher frequency of parkinsonism than DLB females. There were also statistically significant differences in the estimated total intracranial volume, with DLB females showing a smaller intracranial volume as expected.

### Visual rating scales of lobar atrophy (clinical cohort)

We found a significant sex effect on frontal atrophy: the odds for an abnormal score in GCA-F were significantly higher for DLB males (40% had an abnormal GCA-F score) as compared to DLB females (29% had an abnormal GCA-F score, *P*-value = 0.006) (Table 2). There were no statistically significant sex differences in the MTA and PA scales. We did not find a significant interaction between sex and age in any of the three scales.

### Automated estimations of regional atrophy (research-oriented cohort)

**Figure 1:**
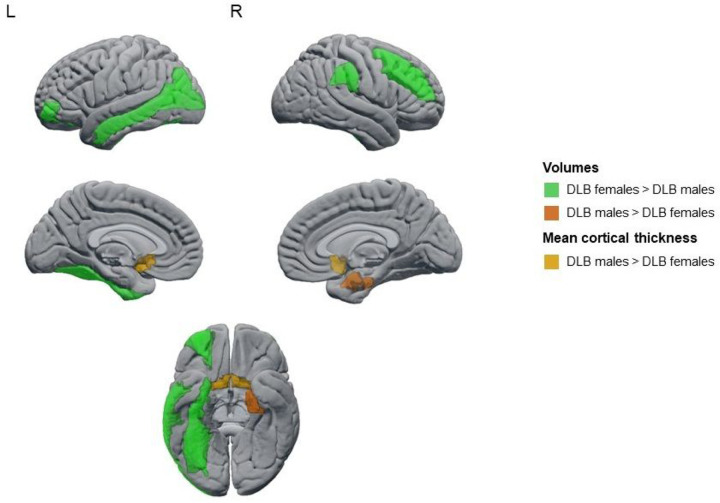
Regions showing statistically significant sex differences in automated estimations of regional atrophy in probable DLB.

We found statistically significant smaller GM volumes in DLB males than in DLB females in the orbital part of the middle frontal cortex, as well as in the middle frontal, fusiform, middle occipital, middle temporal, and supramarginal cortices (Fig. 1, *P*-value ≤ 0.05 in all measures). In contrast, DLB females had a smaller GM volume than DLB males in the right entorhinal cortex, as well as thinner olfactory cortices (Fig. 1, *P*-value ≤ 0.05; Supplementary Table 3).

**Figure 2:**
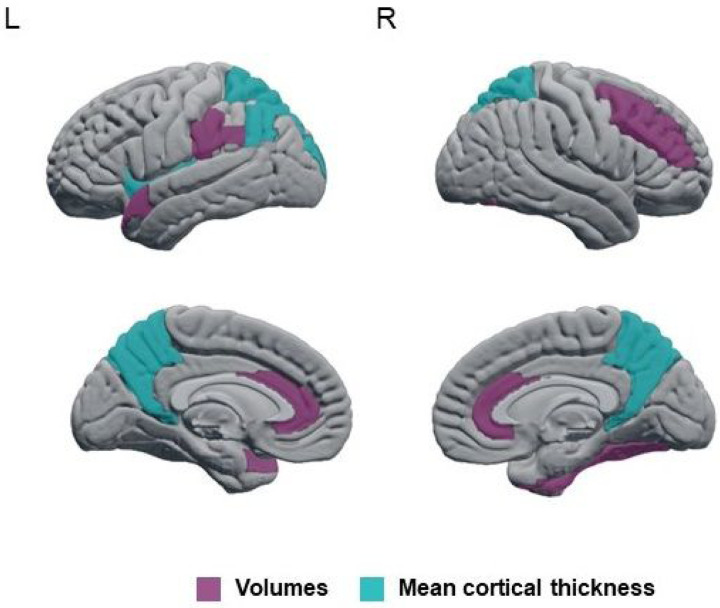
Regions showing statistically significant sex-by-age interactions in automated estimations of regional atrophy in probable DLB.

**Figure 3:**
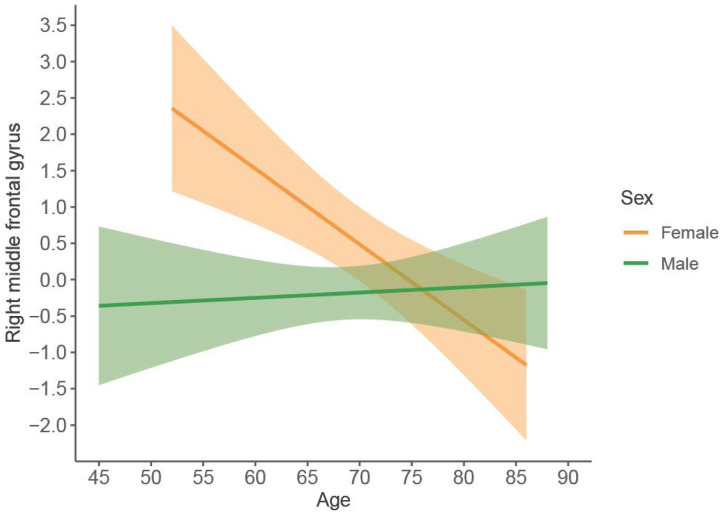
Statistically significant sex-by-age interaction in the right middle frontal cortex. Volume is expressed as residuals obtained from a multiple linear regression model with center and estimated total intracranial volume as predictors. Regression lines depicting female (in orange) and male (in green) probable DLB patients.

We found statistically significant sex-by-age interactions in GM volume in anterior cingulum, middle frontal, fusiform, supramarginal, and superior temporal cortices (Fig. 2, *P*-value ≤ 0.05; Supplementary Table 4). There were also statistically significant sex-by-age interactions in cortical thickness in the angular, insular, superior occipital, and superior parietal cortices as well as in the precuneus (Fig. 2, *P*-value ≤ 0.05; Supplementary Table 4). All these interactions showed that the sex differences in GM were statistically significant at younger ages but tended to no longer be significant at older ages. The interaction for the middle frontal volume remained statistically significant after the FDR adjustment (Fig. 3, FDR-adjusted *P*-value = 0.047).

The sensitivity analyses with MMSE score as an extra predictor showed that male DLB patients had lower left middle occipital and right supramarginal volumes than female DLB patients (*P*-value ≤ 0.05). Further, female DLB patients had lower right entorhinal cortex volume and thinner olfactory cortices than male DLB patients (*P*-value ≤ 0.05). All sex-by-age interactions remained significant after controlling for MMSE (*P*-value ≤ 0.05), including the right middle frontal gyrus (FDR-adjusted *P*-value = 0.043).

Concerning the follow-up models accounting for Alzheimer’s disease co-pathology, *APOE* genotype, and WMH burden, all the models for sex differences remained statistically significant except for the right middle frontal cortex when accounting for *APOE* genotype, and the left fusiform cortex when accounting for WMH burden. The sub-analysis for Alzheimer’s disease status reduced the sample from 165 to 122 participants due to missing data in biomarkers of Alzheimer’s disease. Hence, we first had to replicate our main analyses in the reduced cohort. These new analyses showed sex differences in 4 out of the 9 ROIs with significant sex differences in the 165 cohort, including volume of left middle temporal, right supramarginal, and right entorhinal cortices, as well as thickness of left olfactory cortex (*P*-value ≤ 0.05). Starting the sub-analyses from those 4 ROIs, when we restricted the sample to male and female DLB patients with negative Alzheimer’s disease status (n = 109), the sex differences remained statistically significant for the 4 ROIs (*P*-value ≤ 0.05).

## Discussion

In this study, we investigated sex differences in brain atrophy in probable DLB. Using visual rating scales, we demonstrated the greater frequency of frontal atrophy in male DLB patients as compared with female DLB patients, which may have clinical implications. This finding was replicated in a largely independent cohort using a research-oriented advanced method for automated estimations of volume and cortical thickness. Our analyses also demonstrated that these sex differences tend to disappear with increasing age, with atrophy levels converging in male and female DLB patients after the age of 70. Overall, our findings show a more severe neurodegenerative profile in younger male DLB patients, with no significant sex differences at older ages.

The greater frequency of abnormal scores in the frontal atrophy visual rating scale in male DLB patients replicates the finding from our previous study with a smaller sample (18). Despite using participants from the same E-DLB cohort, the statistical approach and focus differed between the two studies. What we learn from these two studies is that when explicitly testing for sex differences in our current study, male DLB patients have greater frontal atrophy than female DLB patients. In the clinical context, the clinician could expect that almost 40% of male DLB patients would present with frontal atrophy, while female DLB patients would rarely show any frontal atrophy below the age of 70. This indicates that current clinically available cut points for interpretation of frontal atrophy may need to be revisited for DLB (26), and perhaps be redefined by adjusting for both sex and age, as we move towards precision medicine in neurodegenerative disorders. Furthermore, sex may interact with other factors including age, education, disease duration, and CSF biomarkers of AD in driving frontal atrophy (18). These data coming from a large dataset of DLB patients (n = 333) thus encourages to follow up on sex differences in visual ratings of frontal atrophy. Advancing our current understanding of sex differences could optimise interpretations in clinical work up and perhaps enhance the role of structural MRI in the diagnostic criteria of DLB (34), which is currently just supportive due to insufficient sensitivity and specificity values.

We also replicated the clinical MRI results in a largely independent cohort and using a different MRI method, i.e., a research-oriented advanced method for automated estimations of volume and cortical thickness. We found lower GM volume in male than female DLB patients in several frontal, temporal, parietal, and occipital cortical regions. In contrast, female DLB patients showed lower volume in the right entorhinal cortex and thinner olfactory cortices, when compared with male DLB patients. As regards GM estimations of regional atrophy using MRI, only one previous publication explored sex differences in 16 DLB patients (16). The authors found a lower GM volume in male compared with female DLB patients in both frontal and parietal regions. Our study increased statistical power by including 165 DLB patients in the analyses using automated GM volumes. Our findings showed lower GM volumes in male compared to female DLB patients not only in frontal and parietal regions but also in temporal and occipital cortices. On that account, our current findings support a cortical atrophy vulnerability of male DLB patients, not only restricted to anterior cortical regions. While effect sizes were comparable across brain lobes in our study, it seems that visual rating scales can only capture sex differences in frontal lobes, while automated methods may be more sensitive to detect differences across the cortical mantle. Nonetheless, frontal regions were more represented in the findings from the automated method, which may explain the sensitivity of the visual rating scale for frontal atrophy.

Furthermore, the automated method showed that female DLB patients had a lower volume in the right entorhinal cortex. Atrophy in medial temporal regions in female patients could be associated with the higher frequency of Alzheimer’s disease co-pathology previously described in post-mortem studies (13). However, this explanation is unlikely in our cohort because we did not observe any statistically significant sex differences in Alzheimer’s disease pathology. Additionally, we replicated the sex differences in the right entorhinal cortex when we restricted the sample to male and female DLB patients with negative Alzheimer’s disease status. In addition to accounting for Alzheimer’s disease co-pathology, we also adjusted our statistical analyses for *APOE* genotype due to its association with Alzheimer’s disease pathology and atrophy in temporal lobe regions. Since WMH burden also correlates with GM neurodegeneration in DLB (22, 35), we also adjusted our models for WMH. Many of the brain regions with significant sex differences in our main analyses survived the sensitivity analyses for *APOE* genotype and WMH burden adjustments. Altogether, our fundings suggest that the reported sex differences in our cohort are independent of Alzheimer’s disease and cerebrovascualar co-pathologies and *APOE* genotype.

Another interesting finding is the thinner olfactory cortex observed in female DLB patients. Olfactory impairment has been described as a potential hallmark to discriminate between Alzheimer’s disease and DLB (36–38). Notwithstanding, previous studies reported no sex differences in odor identification tests in DLB (36, 38). This suggests a possible discrepancy between function testing and structural neuroimaging studies. A promising prospect for the future would be to investigate differences between male and female DLB patients in olfactory identification in larger samples and elucidate whether their brain structural correlates diverge. This avenue is of further interest to advance our current knowledge about the less investigated ‘olfactory bulb only’ pathological subtype of DLB (39). We recently showed that atrophy in olfactory cortex is one of the brain regions that best discriminates between a DLB subtype with widespread predominant cortical atrophy and a DLB subtype with predominant fronto-occipital atrophy (40). Hence, the interplay between sex, age, and heterogeneity in regional atrophy and clinical phenotype in DLB deserves future investigation.

An important final contribution of this study is the sex-by-age interaction analysis. The most salient finding was the interaction for the volume of the right middle frontal gyrus, which remained statistically significant after the FDR adjustment for multiple testing. In this region, DLB males had similar GM volume across ages, while DLB females showed a steeper slope with lower GM volume at older ages, converging with male DLB patients. This finding expands our previous result, which showed the combined contribution of male sex and older age to frontal atrophy in participants from the same E-DLB cohort (18). In our current study, we demonstrated a statistical interaction and further circumscribed the influence of sex and age to a specific frontal region using a more sensitive method in a largely independent cohort of 165 DLB patients. This finding reinforces the contribution of the male sex to frontal atrophy in DLB observed in the current and previous studies and includes the important consideration about sex differences minimizing as age increases. The shallow slope for male DLB patients in the interaction plots could be interpreted as a plateau level of high atrophy across all sampled ages since 40% of male DLB patients demonstrated abnormal scores in the visual rating scale of frontal atrophy.

We found other regions showing significant sex-by-age interaction in which the male and female patterns resemble the one described for the right middle frontal gyrus. The volumetric results included not only frontal regions (bilateral anterior cingulum) but also temporal (right fusiform gyrus and left superior temporal pole) and parietal regions (left supramarginal gyrus). In addition, the mean cortical thickness analyses showed significant sex-by-age interactions beyond anterior regions, including occipital regions (left superior occipital gyrus), left insular cortex, and parietal regions (left angular gyrus, superior parietal lobules, and bilateral precuneus). Altogether, these findings may reflect a cortical neurodegenerative process initiated at the early or prodromal stages of the disease in male DLB patients and not confined to frontal areas. In this regard, neuroimaging studies on individuals with isolated RBD, a very early phase of alpha-synucleinopathy, show that GM loss is already present in cortical regions (41). Likewise, this finding is coherent with an earlier onset and flatter disease course in male DLB patients, opposite to a more aggressive disease course upon diagnosis in female DLB patients, as proposed by Van de Beek et al. (12). In this regard, future research should address if male patients have an earlier onset of brain atrophy in the prodromal stages of the disease.

The steeper slope of brain atrophy in female DLB patients could be explained by sex-specific neuroprotective factors. Specifically, hormonal levels may promote brain preservation and delayed atrophy in females (42). In this regard, the reduction of estrogen levels (e.g., estradiol) after menopause and with aging may increase the vulnerability to the protein burden and neurodegeneration in female DLB patients. For example, in a study about the effects of menopausal hormone therapy on brain and cognition, we found superior and middle frontal gyri volume preservation after seven years in the treatment group (transdermal 17β-estradiol) compared with the placebo group (43). The maintenance of dorsolateral prefrontal cortex correlated significantly with lower PiB uptake, reflecting lower β-amyloid deposition. This finding highlights the sensitivity of frontal areas to the neuroprotective effect of estradiol in females. This interpretation remains speculative at present but may encourage an exciting line of research on hormonal levels, sex differences, and neurodegeneration in DLB, and neurodegenerative diseases at large.

Finally, we also adjusted our analyses on automated estimations of regional atrophy with MMSE scores as an extra predictor. The results showed that some sex differences remain significant. Male DLB patients had lower left middle occipital and right supramarginal gyri volumes than female DLB patients. Female DLB patients had lower right entorhinal cortex volume and thinner olfactory cortices than male DLB patients. Therefore, the worse global cognitive performance in male DLB patients could be associated with a more anterior (frontal) pattern of atrophy since these regions did not remain significant after controlling for MMSE. In addition, all the sex-by-age interactions remained significant after controlling for MMSE, including the finding in the right middle frontal gyrus.

The main strength of our work is that we demonstrated sex differences in both clinical and research-oriented measures of brain atrophy in two largely independent multi-center DLB cohorts of considerable size. Besides, we analyzed for the first time the interaction between sex and age in brain atrophy, providing findings that may have implications for clinical work up and monitoring of treatment effects in DLB. The main limitation of our study is that the analyses were limited to cross-sectional data. Future studies should investigate the sex-specific trajectories of neurodegeneration in DLB from disease onset or prodromal stages. Longitudinal patterns of atrophy could improve our understanding of the role of sex along the disease course. In this context, the role of sex in DLB heterogeneity should be elucidated, for example aligning with recent studies on disease subtypes based on MRI measures (40).

In conclusion, male probable DLB patients have a more widespread pattern of cortical atrophy than female probable DLB patients, especially in frontal regions. However, these sex-differences minimize with increasing age, especially after the age of 70. These findings may have implications for the interpretation of MRI diagnostic markers in clinical work up, as well as when using MRI as and end point in clinical trials. The characterization of sex differences in DLB from early disease stages is an important future prospect to precision medicine approaches.

## Figures and Tables

**Table 1:** Demographic and clinical characteristics of probable DLB males and females

	*Clinical cohort (N = 333)*				*Research-oriented cohort (N = 165)*			
	Males	Females	n, M/F	t-stat (*P*-value)	Males	Females	*n*, M/F	t-stat (*P*-value)
Age, mean (SD)	72.09 (8.22)	74.74 (8.01)	205/128	**2.885 (0.004)**	68.73 (8.40)	70.02 (9.03)	119/46	0.867 (0.387)
Years of education, mean (SD)	10.89 (3.99)	10.14 (3.78)	171/107	1.567 (0.118)	13.71 (4.02)	13.41 (3.52)	119/46	0.446 (0.656)
MMSE, mean (SD)	22.60 (3.93)	21.92 (4.26)	202/126	1.482 (0.139)	22.33 (5.47)	24.44 (4.18)	119/45	**2.350 (0.020)**
Visual hallucinations (presence)	55.2%	64.4%	174/101	2.221 (0.136)	53.4%	58.7%	116/46	0.366 (0.545)
Cognitive fluctuations (presence)	82.7%	87.5%	110/80	0.816 (0.366)	83.9%	82.2%	112/45	0.068 (0.795)
Parkinsonism (presence)	75.3%	80.7%	166/88	0.945 (0.331)	90.6%	78.3%	117/46	**4.478 (0.034)**
Probable RBD (presence)	78%	50.0%	41/14	0.085 (0.052)	80.2%	71.8%	111/39	1.183 (0.277)
Fazekas scale, *n* 0**/**1**/**2/3	10**/**70**/**30/36	7**/**42**/** 28/21	146/98	7035.0 (0.814)	4/25/10/5	1/6/4/1	44/12	249.5 (0.748)
WMH volume (cm^3^), mean (SD)	*N/A*				16.01 (13.65)	16.39 (12.26)	119/46	0.166 (0.868)
TIV (mm^3^), mean (SD)	*N/A*				1632.00 (134.99)	1441.99 (117.27)	119/46	**8.397 (<0.001)**
AD co-pathology (presence)	14.0%	15.4%	43/13	1.000 (0.603)	10.6%	10.8%	85/37	1.000 (0.598)
*APOE* ε4 carriers (presence)	67.5%	46.2%	40/13	0.200 (0.147)	45.2%	38.6%	115/44	0.561 (0.454)

Statistically significant differences are shown in bold (*P*-value ≤ 0.05).

Abbreviations: F, females; M, males; MMSE, Mini-Mental State Examination; N/A, not applicable; RBD, rapid eye movement sleep behavior disorder; TIV, total intracranial volume; WMH, white matter hyperintensities; AD, Alzheimer’s disease; *APOE*, apolipoprotein E.

**Table 2:** Logistic regression models of visual rating scales

*Model 1: Sex effects*	Effect	*β*	P-value	Abnormal score, *n* (%)	
				Males	Females
GCA-F	Sex	**−2.723**	**0.006**	82 (40.00%)	37 (28.91%)
MTA	Sex	1.160	0.246	75 (36.59%)	55 (42.97%)
PA	Sex	−1.797	0.072	128 (62.44%)	69 (53.91%)
*Model 2: Sex-by-age interaction*	Effect	*β*	*P*-value	Age of abnormal score, mean (SD)	
				Males	Females
GCA-F	Sex*age	−0.634	0.526	74.77 (6.67)	76.84 (6.87)
MTA	Sex*age	−0.942	0.346	73.53 (6.25)	76.78 (6.67)
PA	Sex*age	1.042	0.298	72.45 (8.48)	75.97 (8.19)

Model 1 for sex effects comes from a binary logistic regression model with visual rating scale scores as the dependent variable (normal versus abnormal) and both sex (variable of interest) and age (control variable) as the independent variables. For the MTA scale, the model included age-adjusted score as the dependent variable (normal versus abnormal) and sex (variable of interest). *Model 2* for the interaction between sex and age comes from a binary logistic regression model with visual rating scale scores as the dependent variable (normal versus abnormal) and the interaction between sex and age (variable of interest), together with sex and age as the independent variables. For visual rating scales, values “0” and “1” correspond to “normal” and “abnormal” scores according to established cutoffs. For sex, values “0” and “1” correspond to male and female sex, respectively. Statistically significant effects are shown in bold (*P*-value ≤ 0.05).

Abbreviations: GCA-F, global cortical atrophy frontal-subscale; MTA, medial temporal atrophy scale; PA, posterior atrophy scale.
